# Exploring the Association between Ambient Temperature and Daily Hospital Admissions for Diarrhea in Mopani District, Limpopo Province, South Africa

**DOI:** 10.3390/healthcare11091251

**Published:** 2023-04-27

**Authors:** Zamantimande Kunene, Thandi Kapwata, Angela Mathee, Neville Sweijd, Noboru Minakawa, Natasha Naidoo, Caradee Y. Wright

**Affiliations:** 1 School of Health Systems and Public Health, University of Pretoria, Pretoria 0001, South Africa; 2 Environment and Health Research Unit, South African Medical Research Council, Johannesburg 2090, South Africa; 3 Department of Environmental Health, University of Johannesburg, Johannesburg 2006, South Africa; 4 School of Public Health, University of the Witwatersrand, Johannesburg 2050, South Africa; 5 Applied Centre for Climate and Earth Systems Science, Council for Scientific and Industrial Research, Pretoria 0001, South Africa; 6 Institute of Tropical Medicine, Nagasaki University, Nagasaki 852-8521, Japan; 7 Environment and Health Research Unit, South African Medical Research Council, Pretoria 0001, South Africa; 8 Department of Geography, Geoinformatics and Meteorology, University of Pretoria, Pretoria 0001, South Africa

**Keywords:** apparent temperature, climate change, environmental health, infectious disease, morbidity, threshold regression

## Abstract

Diarrhea contributes significantly to global morbidity and mortality. There is evidence that diarrhea prevalence is associated with ambient temperature. This study aimed to determine if there was an association between ambient temperature and diarrhea at a rural site in South Africa. Daily diarrheal hospital admissions (2007 to 2016) at two large district hospitals in Mopani district, Limpopo province were compared to average daily temperature and apparent temperature (Tapp, ‘real-feel’ temperature that combined temperature, relative humidity, and wind speed). Linear regression and threshold regression, age-stratified to participants ≤5 years and >5 years old, considered changes in daily admissions by unit °C increase in Tapp. Daily ranges in ambient temperature and Tapp were 2–42 °C and −5–34 °C, respectively. For every 1 °C increase in average daily temperature, there was a 6% increase in hospital admissions for diarrhea for individuals of all ages (95% CI: 0.04–0.08; *p* < 0.001) and a 4% increase in admissions for individuals older than 5 years (95% CI: 0.02–0.05; *p* < 0.001). A positive linear relationship between average daily Tapp and all daily diarrheal admissions for children ≤5 years old was not statistically significant (95% CI: −0.00–0.03; *p* = 0.107). Diarrhea is common in children ≤5 years old, however, is more likely triggered by factors other than temperature/Tapp, while it is likely associated with increased temperature in individuals >5 years old. We are limited by lack of data on confounders and effect modifiers, thus, our findings are exploratory. To fully quantify how temperature affects hospital admission counts for diarrhea, future studies should include socio-economic–demographic factors as well as WASH-related data such as personal hygiene practices and access to clean water.

## 1. Introduction

Diarrhea is a leading cause of death and illness and is of particular concern in low- and middle-income countries (LMICs) [1]. According to 2018 estimates, approximately 1.6 million deaths occur each year globally due to diarrhea [2,3]. Annually, it is estimated that among children younger than 5 years, there are 1.7 billion cases of diarrheal disease and between 370,000 and 525,000 diarrhea-related deaths [4,5]. The incidence of childhood diarrhea is highest in LMICs, with Africa and Asia bearing the largest burden of the disease [6]. There are several risk factors for diarrhea, which include: low economic status, a lack of education, poor water storage practices, not treating drinking water, and overcrowding [7,8]. Environmental conditions can also affect the incidence of diarrhea by influencing factors such as the transport, diffusion, reproduction, and persistence of certain pathogens that cause diarrhea [9,10]. For example, ambient temperature may impact the survival of the bacterial pathogens and or host behaviour. The agents causing diarrhea seem to multiply rapidly and survive for longer in warmer conditions [11]. Previous studies show that increases in daily ambient temperature are associated with higher numbers of hospital admissions for both adult and childhood diarrhea [1,2,3,4,5,6,7,8,9,10,11,12,13,14,15,16,17]. Risk of diarrheal disease has also been found to increase as humidity increases [18]. These studies suggest the significant role of temperature and humidity in influencing diarrheal patterns. Therefore, it is important to consider indicators that combine meteorological variables, such as apparent temperature.

In South Africa, diarrhea is common, especially among children under 5 years of age. There is an estimated prevalence of 13% in this age group for the country that was reported in a nationally representative study from 1998. (This is relatively old, but more recent data are unavailable) [19]. Few studies in South Africa have considered the relationship between temperature and diarrhea. One described the relationship between minimum and maximum temperature and childhood diarrhea cases seen at healthcare facilities in Cape Town [20]. A 5 °C increase in minimum and maximum weekly temperatures led to a 40% and 31% increase in diarrhea cases in the first and second summer season during the study period, respectively [20]. Furthermore, a rural-based study explored the relationship between temperature, precipitation, and diarrhea-related hospital admissions in Limpopo province using contour analysis [21]. Findings suggested that children under five years of age were most vulnerable to diarrhea during very dry, hot conditions. However. there were no statistically significant associations between temperature and diarrhea cases in people aged five years and older [21]. Moreover, the type of analysis used did not quantify the effect of temperature on diarrhea morbidity between the age groups, nor did it provide thresholds for the temperature–admission relationship.

To consider the effect of temperature on diarrhea morbidity on various age groups mentioned above and possible threshold temperatures for the temperature–hospital admission relationship, this study aimed to investigate the relationship between daily average temperature and total daily diarrhea-related hospital admission counts in Mopani district, Limpopo province, from 2007 to 2016 for (i) individuals of all ages; (ii) children aged five years and under; and iii) individuals over the age of five years (iii).

## 2. Materials and Methods

### 2.1. Study Design

**Figure 1:**
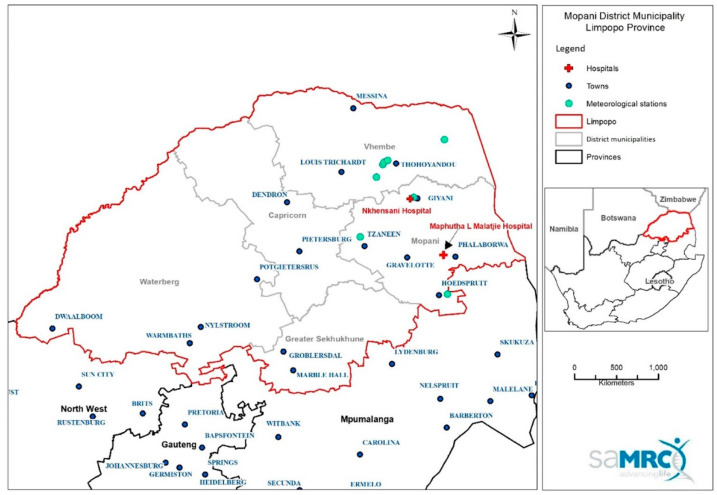
Location of the two hospitals and several meteorological stations in Mopani, Limpopo province, South Africa.

The study comprised a retrospective analysis of daily hospital admission records and daily ambient average temperatures. Furthermore, the study’s area included the two largest local municipalities, namely, Giyani and Phalaborwa within the largest district, Mopani district municipality in Limpopo province (Figure 1) [22]. Additionally, the two largest government hospitals in these municipalities in Limpopo were selected to try and obtain maximum number of hospital admissions for diarrhea in the area. Data from both hospitals were combined in one dataset to try and represent the geographical area of interest. Since we were aware after a site visit that the data were not digital and would need to be manually captured at a sizeable cost, our budget limited us from including other hospitals in the province.

### 2.2. Daily Hospital Admissions Data

Data were retrieved from handwritten hospital admission record books by first scanning admission record books’ pages that were saved in PDF format. Following this, data were then manually captured into electronic format using Epi Data software [23]. No ICD-10 codes were available in the hospital admissions records. Diarrhea cases were extracted from the hospital admission records database using the terms identified in the dataset and confirmed by a South African medical doctor. All the terms used to identify diarrhea admission cases within the dataset are provided in Supplementary Table S1. We included diarrhea, gastroenteritis, dysentery, and acute gastroenteritis with abdominal pain. The number of cases included was 9956. There were 33 excluded cases for ‘abdominal distention without mention of diarrhea’, since abdominal distention could be associated with a variety of medical conditions other than diarrhea. Therefore, the final provided total daily hospital admission counts for diarrhea were *n* = 9923.

Data variables for daily hospital admissions included the age and gender of the patient, however, the gender data were poorly completed and not included in this study. Individual, daily hospital diarrhea admissions were aggregated to daily totals/counts. We decided to create three groups of daily hospital admissions for diarrhea: (1) for individuals of all ages, in other words, all diarrhea admissions; (2) for children aged 5 years or younger; and (3) for individuals older than 5 years. The rationale for this was the high reported prevalence of diarrhea among children aged 5 years or younger [24]. Thus, we could explore whether daily average temperature was associated with daily diarrhea hospital admissions in this specific group.

### 2.3. Meteorological Data: Temperature, Relative Humidity, and Wind Speed

Ambient temperature data, relative humidity, and wind speed were obtained with permission from the South African Weather Service (SAWS) for the same period as the daily hospital admissions dataset. Daily average temperature, daily minimum temperature, and daily maximum temperature, as well as daily relative humidity and daily average wind speed, were provided by SAWS for two weather stations in the study area: Giyani and Thohoyandou. The Giyani weather station dataset had missing data, while the Thohoyandou weather station had no missing data. Hence, we used the Thohoyandou meteorological datasets in this study. In a previous study [25], temperatures measured at the Thohoyandou weather station and in dwellings in Giyani (near the Nkhensani Hospital), were well-correlated (R = 0.98, *p* < 0.0001), suggesting that meteorological conditions did not vary substantially between the Thohoyandou station and the communities living nearby the hospitals in the study.

### 2.4. Apparent Temperature (Tapp)

Daily average temperature from SAWS was used to calculate apparent temperature (Tapp). Tapp is an adjustment to the ambient temperature based on the level of relative humidity and wind speed. It is also considered a measure of how humans perceive or feel temperature and was selected for this study. Moreover, since Tapp is used to gauge likely human physical reactions to weather conditions, including to examine the association between human health and exposure to temperature, it was also included in this study [26,27].

Previous studies considered the relations between meteorological variables, e.g. daily temperature, relative humidity, rainfall, and diarrhea, (either as individual variables or combined in various forms), including Tapp [28,29,30]. Tapp has also been applied for other health outcomes such as cardiovascular diseases and respiratory diseases [31,32,33]. Therefore, we followed a similar approach. Daily Tapp was calculated using the equations below [26]:

AT=Ta+0.33×e−0.70×ws−4.00

where:

*Ta* = dry bulb temperature (°C);

*e* = water vapor pressure (hPa);

*ws* = wind speed (m/s) at an elevation of 10 m.

e=rh/100×6.105×exp(17.27×Ta/(237.7+Ta))

where:

*rh* = relative humidity (%)

### 2.5. Statistical Analyses

First, distributed non-linear lag modelling (DLNM) was applied to the data. However, due to low daily counts and missing data, there was a lack of statistical power. The methods and results from the DLNM analyses are included in Supplementary File S2.

Linear regression was used to examine the associations between daily hospital admissions for diarrhea and (i) daily average temperature, and (ii) daily Tapp. The estimated changes in hospital admissions per unit increase in temperature (°C) were reported with their associated 95% confidence intervals (CIs), and *p* < 0.05 was considered statistically significant. Analysis was performed for all individuals in the hospital admissions dataset; admitted for diarrhea to consider the overall association and then analysis was stratified by age. Moreover, hospital admissions for diarrhea were categorized into two age groups. The groups were as follows: children five years of age and younger, and individuals older than five years of age.

Threshold regression was applied to determine thresholds for daily average temperature. Daily Tapp for associations that were statistically significant in the linear regression model were also applied. Threshold regression models are a class of regression models that estimate associations between predictors and outcomes in a threshold-dependent way. The threshold parameter is also known as a change point. This refers to the point at which the relationship between the outcome and the predictor differs. Threshold regression extends linear regression and detects structural changes along a natural axis, and allows coefficients to differ across two regions [27]. The results are presented as a threshold value, which is the change point identified by the model, and coefficients for each region. Region 1 represents the portion of temperature values less than or equal to the threshold. Region 2 corresponds to temperatures above the threshold. The coefficients for each region signify the amount of change in hospital admissions above and below the threshold, and the direction of the association between x and y variables (positive or negative).

Results are presented as estimates of daily average temperature and daily Tapp thresholds, the coefficients on either side of the thresholds and the associated 95% CIs and significance level. All statistical analyses were conducted in STATA version 15.0 [34].

## 3. Results

**Figure 2:**
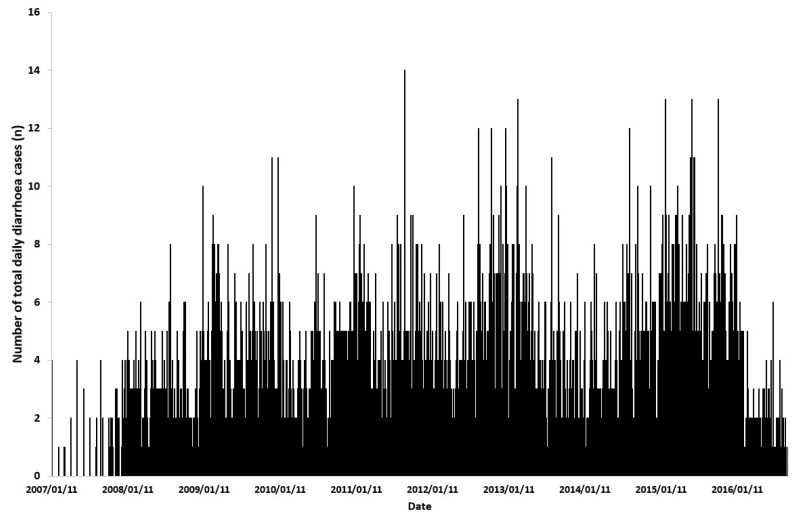
Total daily number of hospital admissions counts for diarrhoea for the study period 2007 to 2016.

About one-third of total daily hospital admissions were for diarrhea among individuals of all ages (Table 1). The mean daily total number of hospital admission diarrhea cases was 3 (range 1 to 14). The largest proportion (64%) of total daily diarrhea hospital admission cases were among children aged 5 years and younger. Figure 2 shows the total daily number of diarrhea hospital admission counts over time for all individuals admitted with diarrhea during the study period. There were missing data in 2007.

### 3.1. Meteorological Findings

Daily average temperature and Tapp range between 2–42 °C and −5–34 °C respectively, showing some differences between the minimum and maximum values for these two variables. (Table 2).

**Figure 3:**
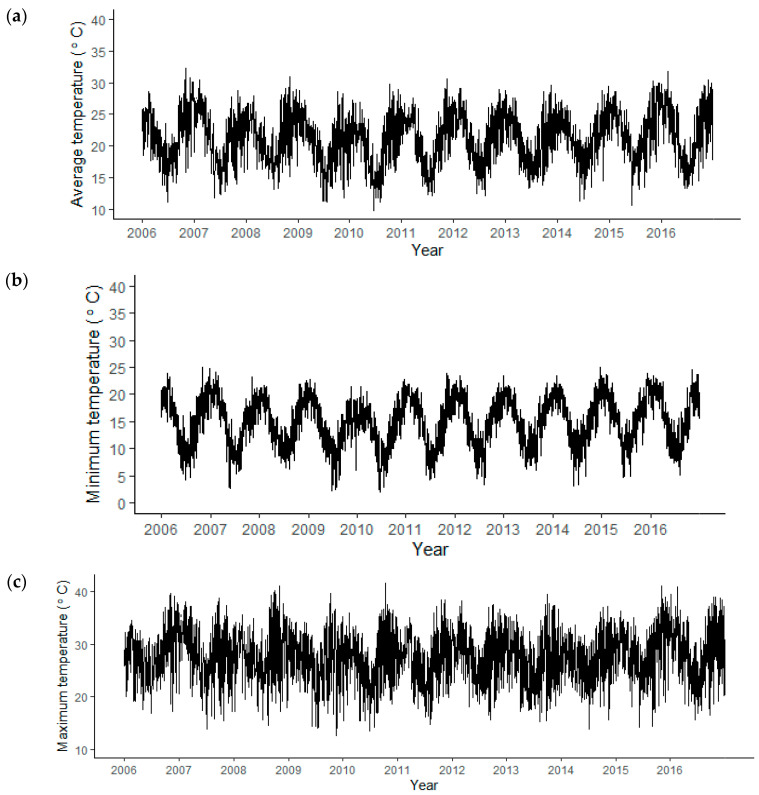
Trends in (**a**) average, (**b**) minimum and (**c**) maximum temperature (°C) for 2007 to 2016.

Ambient daily average, minimum and maximum temperature show typical seasonal trends of summer and winter variations with warmer and cooler average temperatures in these seasons, respectively (Figure 3). Daily minimum temperatures show slightly less variation compared to average and maximum daily temperatures.

### 3.2. Relationship between Daily Average Temperature, Tapp, and Daily Diarrhea Hospital Admission Counts

Daily average temperature and Tapp have significant positive linear relationships with the total number of daily diarrhea hospital admission counts for individuals of all ages (Table 3). For every 1 °C increase in average daily temperature, there is a 6% increase in hospital admissions for diarrhea for individuals of all ages, and a 4% increase in admissions for individuals older than 5 years. These findings, therefore, are statistically significant.

Results are similar for average daily Tapp, although %-increases are smaller compared to those for average daily temperature. For every 1 °C increase in average daily Tapp, there is an increase of 2% in admissions of diarrhea for individuals of all ages. Additionally, there is a 2% increase in the grouped category of individuals older than 5 years old. There are no statistically significant trends for children aged 5 years and younger for daily average temperature, or average daily Tapp and hospital admissions for diarrhea, although the relationships are positive.

#### Threshold Regression Results

Threshold regression was conducted on associations that were statistically significant in the linear regression. Hence, children aged 5 years and younger were not included. In the group of individuals older than five years, daily diarrhea admissions show a statistically significant increase in admissions; with daily average temperature above 16.3 °C. For the same age group, daily Tapp ≤ 14.5 °C is associated with reduced diarrhea admissions (*p* = 0.009). There is also a statistically significant increase for diarrhea hospital admissions when daily average Tapp ≤ 26.5 °C for individuals of all ages admitted with diarrhea (Table 4) are analyzed.

## 4. Discussion

This study assessed the association between daily ambient temperature and daily hospital admissions for diarrhea in a rural South African setting. We considered both daily average temperature and daily average Tapp, (a real-feel temperature often used in similar studies to ours) [32,35], in relation to daily hospital admissions for diarrhea. Individuals of all ages and two grouped categories: children ≤5 years, and individuals >5 years, were used in this study. Statistically significant positive associations between daily average temperature and daily average Tapp were found, as well as diarrhea-related hospital admissions for two of our three grouped categories, mentioned above. For example, individuals of all ages include the elderly. Thus, the need for adequate hospital treatment and care is essential.

There is little difference when comparing our findings for temperature and Tapp. Tapp is temperature ‘modified’ by relative humidity and wind speed. In contrast, our study area is an inland site with relatively low relative humidity and low wind speed. Thus, the Tapp values are generally lower than temperature values. We do not see a statistically significant association between daily average temperature, or daily average Tapp and daily admissions for diarrhea for children aged 5 years and younger. At first, we thought this was surprising, since other studies noted this effect in children aged 5 years and younger—although the age ranges in the study do differ [20,36,37,38,39].However, there are several possible reasons for this finding. Many non-climatic risk factors are associated with diarrhea [40,41] among children aged 5 years and younger. Therefore, it is unlikely that temperature on its own would be found to be associated with diarrhea among these children. Studies in LMICs have found that low economic status, a lack of maternal/paternal education, poor water storage practices, not treating water, poor sanitation and overcrowding all influence diarrhea prevalence among children aged five years and younger [10,11]. Floods and drought have also been associated with the occurrence of diarrhea, including among children five years of age and younger [42,43,44]. Data from demographic health surveys across sub-Saharan Africa found weak associations between temperature and cases of diarrhea in children under five years. However, a shortage of rainfall in the dry season increased the prevalence of diarrhea across sub-Saharan Africa [44].

We found that daily diarrhea admissions for all individuals increase when daily Tapp is equal to or below 26.5 °C. For individuals over five years old, an increase in diarrhea admissions was predicted when daily average Tapp was above 16.3 °C. A similar threshold was observed in China. Diarrheal disease risk in the age group 20 years and older increased when mean Tapp rose above 15 °C [45]. A decrease in admissions was estimated when Tapp was equal to or below 14.5 °C. Threshold values for daily average Tapp were similar to those for daily average temperature and those seen in China—where hospital admissions for diarrhea increased by 1.06% for every 1 °C above 12.5 °C [46]. In our study, the higher temperature threshold identified for the group of individuals of all ages admitted for diarrhea, is likely due to the large age range in this group from 6 years to older than 60 years. One might explore the data with age group categories of smaller sizes to identify vulnerable groups, in future work.

### Limitations

All the hospital admission records were handwritten and posed numerous challenges such as faded ink and handwriting being illegible; the daily use of books leading to torn or missing pages. In almost all cases, the gender was missing. Therefore, it was not possible to use these data in our analyses. Despite those hurdles, the results presented here are statistically significant, and missing and incorrect reporting are unlikely to drastically change the conclusions. There was no ICD-10 code assigned to the reason for admission. This was why we provided all the terms from the hospital admissions books that we used to define a diarrhea case, after we had verified these terms with a medical doctor.

We used regression and threshold analyses to try and accommodate low daily hospital admissions data, for diarrhea and missing data (particularly in 2007). These analyses do not permit controlling for day of the week, or holidays, and do not consider lag effects. We were unable to include total the population in the analysis, because we did not have the home addresses of admitted individuals to be able to assign them to a geographical region with known population estimates. We acknowledge that this was an exploratory analysis, and should consider other robust statistical techniques for a larger, more comprehensive, and complete dataset.

The daily hospital admissions data were from two of the largest hospitals in the largest district in the province; however, it is likely that not all cases of diarrhea in the community resulted in hospital admissions. So, the data might not be representative of the scale of the diarrheal prevalence. Also, in rural areas, some residents prefer to seek medical advice from traditional healers instead of reporting to health facilities such as clinics or hospitals. Diarrhea may also be treated symptomatically with over-the-counter medicine, and only complicated cases present at the hospital [47].

Unfortunately, hospital admissions for diarrhea did not have matching laboratory data for stool samples taken to confirm the presence of a diarrheal pathogen. Laboratory confirmation of admission cases for diarrhea was therefore not possible. It is suspected that the increased number of diarrhea cases that were observed during warmer weather may be attributed to enteric bacteria, as seen in other studies [48,49,50,51,52]. However, this cannot be confirmed. This is a motivating reason for a shift away from handwritten admissions books, and to connect hospital admissions, samples, and laboratory data, so that the best quality research can be performed, which includes conducting a sensitivity analysis for cases caused by rotavirus, for example.

The interpretation of our findings is constrained—since models could not include potential co-variables and confounding factors that affect diarrhea, such as demographics, susceptible sub-population groups, and socio-economic status. These data were not captured in the hospital records. Many of these factors lead to lack of access to clean water that can decrease water for domestic use. Hand washing, as well as for the cleaning of outdoor pit latrines, and reduce personal hygiene and sanitation quality are a few examples. However, we did not have information about household water source and water quality for each diarrhea case, Furthermore, the absence of residential addresses or contact numbers, meant it was impossible to follow-up with individuals. It is known that several South African communities are vulnerable to diarrhea because they are impoverished. South African children from underprivileged families are reported to be ten times more probable to die from diarrhea than children from relatively privileged families [9,53]. Malnourishment, poor environmental conditions, and conditions such as HIV/AIDS, cause children to be more vulnerable to severe diarrhea and dehydration [54]. While access to water has improved in South Africa, safety, reliability, convenience, and access to clean water, are still important concerns. Some households continually rely on water from untreated sources, such as rivers, dams, and boreholes [50,51]. Limpopo is one of South Africa’s provinces with the lowest percentage of access to clean water supplies. Households in Limpopo with access to treated, piped water, face water interruptions leading to the use of untreated water. Sources include: rain, water retail trucks, rivers, boreholes and springs [55,56]. In the case of water interruptions and shortages, storage of water occurs. Incidence of acute diarrhea has been shown to result from the presence of fecal coliforms in water; stored in containers in rural households [56]. We acknowledge that rainfall and water usage/storage play an important role in diarrhea prevalence. Future studies with more complete datasets should consider modelling both temperature and rainfall, as well as other potential co-variates and confounders.

## 5. Conclusions

Daily average temperature was associated with increased risk of hospital admissions for diarrhea. However, this research was exploratory, and the findings should be interpreted with caution. Future studies that consider meteorological and climatic factors’ influence on diarrhea hospital admissions, should aim to include a broad range of potential confounders and co-variables (at both the individual and household level).

## Figures and Tables

**Table 1:** Total number (N) of daily hospital admissions and daily number (*n*) of cases of diarrhea by individuals five years and under, and over five-year-olds for 2007 to 2016.

Variable	Frequency of Cases	Percentage of All Admissions (%)	Percentage of Diarrhea Admissions (%)
All hospital admissions (N)	30,784	-	-
Cases of diarrhea in all ages (*n*)	9923	32	-
Cases of diarrhea in children ≤5 years (*n*)	6362	21	64
Cases of diarrhea in individuals >5 years (*n*)	3561	12	36

**Table 2:** Summary of daily meteorological data during the study period of 2007 to 2016.

Variable	Average	Minimum	Maximum	5th Percentile	95% Percentile
Temperature (°C)	21	2	42	15	27
Relative humidity (%)	65	39	85	36	89
Wind speed (m/s)	3	0	32	2	28
Apparent temperature (°C)	21	−5	34	12	28

**Table 3:** Results of linear regression showing the association between daily hospital admissions (*n*) for diarrhoea, temperature (°C) and Tapp (°C).

	Hospital Admissions for Diarrhoea	Estimates * (β)	95% CI	*p*-Value
Average daily temperature (°C)	All admissions	0.06	0.04–0.08	**<0.001**
	Over 5 years	0.04	0.02–0.05	**<0.001**
	5 years and younger	0.01	−0.00–0.03	0.107
Average daily Tapp (°C)	All admissions	0.03	0.02–0.05	**<0.001**
	Over 5 years	0.02	0.01–0.04	**0.001**
	5 years and younger	0.01	−0.00–0.02	0.110

Note: * Estimates can be converted into a percentage using the formula (exp(β) − 1) × 100. Bold indicates statistically significant relations.

**Table 4:** Model estimates from the results of the threshold regression model for diarrhoea hospital admissions (*n*) of children aged 5 years and under, and for admissions (*n*) over 5 years of age.

Hospital Admissions for Diarrhoea	Threshold Variable	Threshold Value °C	Region	Model Coefficient	95% Confidence Interval	*p*-Value
All admissions (all ages)	Average daily temperature	23.0	Region 1	0.026	−0.01–0.06	0.13
			Region 2	0.027	−0.05–0.11	0.49
	Average daily Tapp	26.5	Region 1	0.018	0.001–0.03	**0.04**
			Region 2	0.018	−0.15–0.18	0.82
Admissions over 5 years of age	Average daily temperature	16.3	Region 1	−0.030	−0.19–0.13	0.70
			Region 2	0.025	0.01–0.05	**0.03**
	Average daily Tapp	14.5	Region 1	−0.104	−0.18–(−0.02)	**0.01**
			Region 2	0.011	−0.01–0.03	0.19

Notes. The admissions for 5 years and younger were not included here since the results were not statistically significant in the linear regression, hence they were not carried forward into the threshold regression. Region 1 corresponds to the average temperature and Tapp values less than or equal to the respective thresholds (column 3). Region 2 corresponds to average temperature and Tapp values greater than the respective thresholds (column 3). Bold *p*-values indicate statistically significant results.

## Data Availability

Data are available upon request from the corresponding author.

## Supplementary Materials

The following supporting information can be downloaded at: https://www.mdpi.com/article/10.3390/healthcare11091251/s1, Supplementary File S1: Table S1—Terms from the hospital admission books used to identify cases of diarrhea for this study. Supplementary File S2: Figure S1—The estimated effect of the association between mean temperature and diarrhea admissions in Mopani District shown by (a) three-dimensional plot and (b) contour plot.; Figure S2—Lag effects for specific temperatures and lag periods of diarrhea-related hospital admissions in Mopani District.; Figure S3—The (a) lag-response curves for specific temperatures, i.e., 20 °C, 15 °C, 25 °C, and 27 °C, in Mopani District and (b) overall cumulative effect. References [57,58] are in Supplementary Materials.
