# Supplementary material for: Exploring the Association between Ambient Temperature and Daily Hospital Admissions for Diarrhea in Mopani District, Limpopo Province, South Africa
Source: Healthcare (Basel). 2023 Apr 27;11(9):1251. doi: 10.3390/healthcare11091251 (PMC10177752; doi:10.3390/healthcare11091251)
Supplement: Supplementary file 1 [file healthcare-11-01251-s001.zip › healthcare-2251154-supplementary.pdf]

Supplementary File S1

Table S1. Terms from the hospital admission books used to identify cases of diarrhoea for this study.

-ge<br>-gastro<br>-gastroenteritis<br>-gastro-enteritis<br>-gastro enteritis<br>-gastro acute enteritis<br>-AGE (acute gastro enteritis)	-diarr<br>-diar<br>-diarrhoea<br>-chronic diarrhoea<br>-severe diarrhoea<br>-diarrhea<br>-chronic diarrhea<br>-severe diarrhea<br>-acute diarrhea<br>-acute diarrhoea<br>-diarrhoeal infection<br>-inflammatory diarrhoea	- dysentery

Supplementary File S2: Distributed Non-linear Lag Analysis Methods and Results

Methods

The temperature-diarrhoea admissions relationship was analysed using distributed lag non-linear model (DLNM) with natural cubic spline (NCS) as its smoothing parameter applied to both average temperature and lag dimensions. Involved inside the initial model were variables such as the predictable value of the daily counts of mortality (Yt), which follows an over-dispersed Poisson distribution, vector of regression coefficients for the cross-basis (β), cross-basis in pre-determined temperature and lag dimensions (Tt,l), seasonal variations (date), day of the week (dow), and day of the year (doy).The statistical approach focussed on a quasi-Poisson model within Generalized Linear Model (GLM) and used a Poisson distribution of daily diarrhoea admissions while accounting for over-dispersion and autocorrelation [Gasparini et al., 2010].

This methodology is based on a "cross-basis" function that describes a bi-dimensional association along the dimensions of temperature and lag days, which not only allows for examination of the relationships between temperature and diarrhoea admissions at each lag period but also allows for the estimation of non-linear effects across lags [Gasparini et al., 2010].

Two independent functions were defined in the space of the predictor and in the space of the lag as exposure-response and lag-response, using specific functions for each function, and specific knots for the spaces of both exposure- and lag-response functions. The two functions were combined in one cross-basis function describing a bi-dimensional relationship which represents a complex dependency of the association in the space of the predictor temperature and the outcome (diarrhoea admissions) within the space of lag-responses. A maximum delay of 30 days according to previous studies was chosen to capture the full lag pattern of both hot temperature (harvesting) and cold temperature (prolonged effect). The study assumed the bi-dimensional exposure-lag response allowing the time-varying nonlinear and delayed effects of the Tavg on diarrhoea admissions in the Mopani District [Gasparini, 2011].

The cross-basis function was incorporated thereafter in GLM using the quasi-Poisson regression for both the case cross-over and time series designs. The complex dependency of bi-dimensional effect of exposure-lag response on diarrhoea admissions highlights Tavg delayed and nonlinear effects in time by using the DLNM framework. The DLNM in the cross-basis was parameterized using smooth functions such as the natural cubic spline ("ns") function in the space of the predictor with 3 knots, followed by a linear function ("lin") in the space of lag response using 3 knots. In the space of the variable 5df was used and 3df in the space of lag and all the knots were equally spaced in the space of the predictor-response as well as in the space of the lag-response. The bi-dimensional effect within the cross-basis function was applied along lag interval of 0 as the minimum lag up

to 30 days as the maximum lag assuming past exposure to $Tavg_{x-l}$ to explain diarrhoea admissions at a given time day_t.

A Poisson regression model was used to permit for over-dispersion, to combine the case–crossover design with a DLNM and to evaluate the area-specific associations among the daily average temperature and daily admissions of diarrhoeal diseases for population of all ages. DLNM analysis allows for a non-linear dependency with temperature by adopting a bi-dimensional standpoint to represent associations which vary non-linearly along the space of the predictor and lags [Gasparini, 2011].

From time to time the result of an exposure incident is not limited to the period when it is experienced, but later in time. This makes it difficult to validate the association between an exposure incident and the order of future outcomes, specifying the spreading of the effects at different times after the occurrence (known as lags). Due to this fact additional lag dimensions of an exposure–response need to be described. This is usually seen on short-term exposures.

The sensitivity analysis was done with the purpose of selecting the best close-fitting model in the case cross-over and time series models. The selection criteria were grounded on models with the lowest Akaike Information Criteria (AIC) and lowest Partial Auto-Correlation Function (PACF). The two functions, AIC and PACF, were used to see model fit and to maximize the ability of the model to predict the best exposure-lag response relationship. The AIC for quasi-Poisson models allowed for use of natural cubic splines with 5df for the daily mean temperature and 3df for lag stratification produced the best model fitting. In the final model, 7df per year to control for seasonality and long trend components.

A maximum lag of 30 was assumed to completely capture the overall area-specific temperature effects on diarrhoea admissions and adjust for any potential harvesting. The relative risk (RR) of daily average temperature on diarrhoea admissions along lag days was examined, with the median value of area-specific temperature distribution as the baseline reference value. The RRs were plotted against temperature and lags to show the entire relationship between daily mean temperature and diarrhoea admissions in the Mopani District, and the cumulative 30-day overall effect of temperature on diarrhoea admissions was also plotted. Then the temperature–diarrhoea association between daily mean temperature was compared to identify the characteristics of exposure–response relationships when different temperature indices were chosen.

The specific model was:

$$Y_t \sim \text{Poisson}\ (\mu_t) \quad \text{(S1)}$$

$$\text{Log}\ (\mu_t) = \alpha + \boldsymbol{\beta} t_{,t,l} + ns\ (time,\ 7*15\ ) + as.factor(dow) + as.factor\ (doy) \quad \text{(S2)}$$

where t indicates the day of observation, Y_t is the observed diarrhoea counts on day t, α as an intercept expected, $t_{,t,l}$ is a matrix obtained by applying the DLNM to temperature, $\boldsymbol{\beta}$ is the vector of coefficient for $t_{,t,l}$ and l is the lag of days. The natural cubic spline of time, denoted as ns (time, 7*15), to control for the effects of seasonality, long-term trends, day of the week (dow) and day of the year (doy).

Results

The model was fitted using a cubic b-spline and three equally space knots and 3df for mean temperature, a natural cubic spline with 5df for the lag space, and a natural cubic

spline with 7df per year was applied, adjustments were included for days of the week, and day of the year (Figure S1).

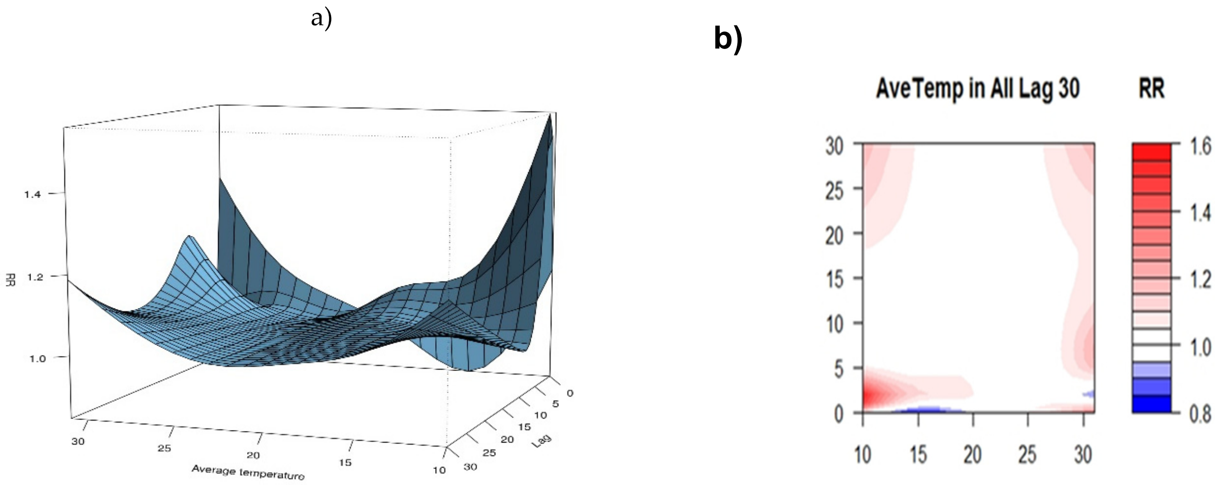

Figure S1 The estimated effect of the association between mean temperature and diarrhoea admissions in Mopani District shown by a) 3-dimensional plot and b) contour plot.

The 3-dimension plot shows the relationship between temperature and diarrhoea admissions along lag days (Figure S1). The temperature effects were non-linear. Higher risks were associated with both hot and cold temperature. An acute increase in risk was observed for cold temperatures; the diarrhoea admissions increased sharply in the first 5 days at 10°C in contrast to the same day exposure to extremely hot temperature.

Figure S2 shows the bi-dimensional RR surface for diarrhoea hospital admission using a reference value of 22°C: the dashed lines represent the effects by lags for specific temperatures (10°C and 27°C) and conversely the effects by temperature at specific lags (9 and 15 days).

Lag-specific effects were investigated to assess the relationship between diarrhoea-related hospital admissions and temperature. Figure S2 shows the relative risks of diarrhoea admissions associated with temperature at lag 9 and lag 15. It also shows association between risks of admission and lag periods at low (10 °C) and high (27 °C) temperatures.

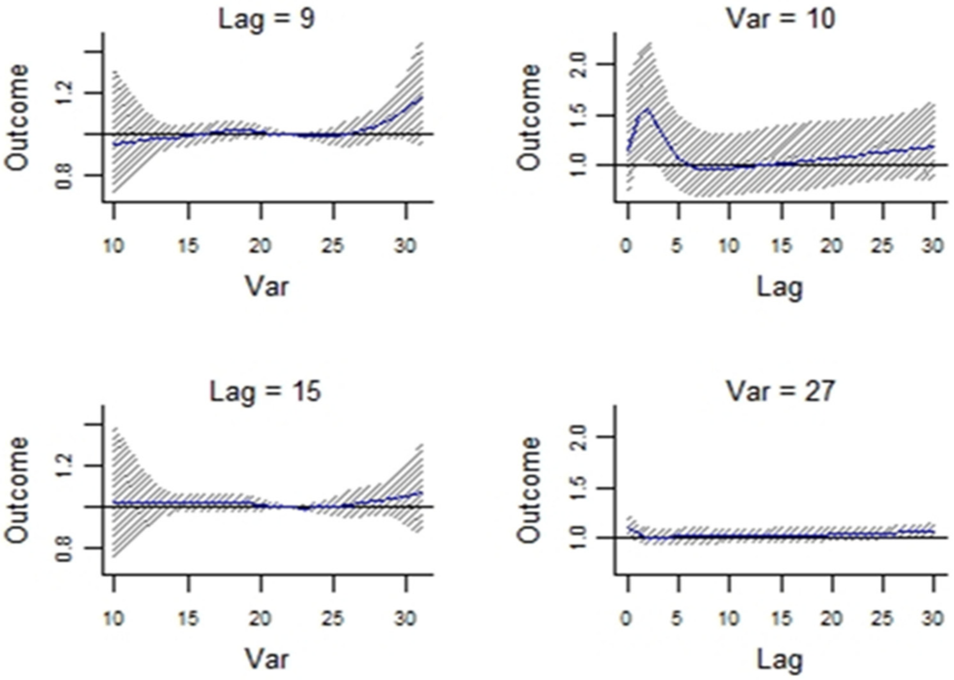

Figure S2 Lag effects for specific temperatures and lag periods of diarrhoea-related hospital admissions in Mopani District.

The results from this model propose an effect of cold in the first 5 days, followed by a reduction after around 5 days, possibly taken as harvesting; hot temperatures displayed a more delayed effect, lasting up to 15 days. Figure S3 shows the pooled cumulative temperature-mortality association over lag 0–30 days projected by the multivariate meta-analytical model. The solid line signifies RRs and the shaded area is the 95% CI. The vertical solid line and the dashed lines signify the minimum diarrhoea admissions temperature and the 1st (10°C), 25th (18°C), 75th (25°C), and 99th (29°C) percentiles of the national-level average temperature distribution, correspondingly.

Summary

DLNM was used to evaluate temperature effects on diarrhoea admissions. The lag days of both hot and cold temperature were evaluated and showed immediate cold effect and longer hot effects.

A U-shaped association was observed between temperature and diarrhoea admissions. These results suggested that both minimum and maximum temperatures were associated with a rise in diarrhoea admissions. The effects of lower temperature appeared acute, while the effects of higher temperatures were late. Cold temperatures were associated with higher risk for diarrhoea admissions at lag 0-5; the immediate cold effects were followed by negative estimates lasting for about one week which is consistent with the theory of morbidity displacement and the harvesting effect. The highest pooled overall RR was RR = 8 (95% CI: 0.9 - 70.4) however the 95% CI was large.

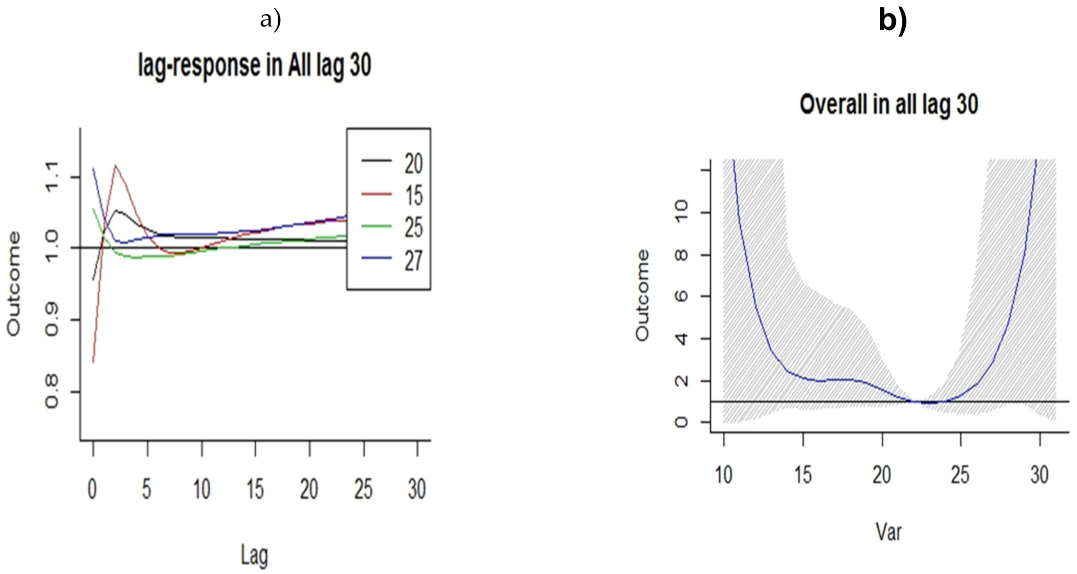

Figure S3 The a) lag-response curves for specific temperatures, i.e., 20°C, 15°C, 25°C and 27°C, in Mopani District and b) overall cumulative effect.

We found there was a major limitation to applying DLNM to the dataset. Substantial missing data, including all of 2006 and most of 2002 and 2004 affected statistical power as the daily counts were very low. Rather than abandoning the data, we decided to apply linear and threshold regression, despite its shortcomings, to further explore the data.

References

1. Gasparrini A, Armstrong B, Kenward MG. Distributed lag non-linear models. Statistics in medicine. 2010;29(21):2224-34.
2. Gasparrini A. Distributed lag linear and non-linear models in R: the package dlnm. Journal of statistical software. 2011;43(8):1.
